# Inflammatory processes and schizophrenia: two independent lines of evidence from a study of twins discordant and concordant for schizophrenic disorders

**DOI:** 10.1007/s00406-017-0792-z

**Published:** 2017-04-04

**Authors:** Silke Braun, René Bridler, Norbert Müller, Markus J. Schwarz, Erich Seifritz, Matthias Weisbrod, Alexandra Zgraggen, Hans H. Stassen

**Affiliations:** 1 Psychiatric University Hospital (KPPP), Institute for Response-Genetics, University of Zurich, 8032 Zurich, Switzerland; 2 Sanatorium Kilchberg, 8802 Kilchberg, Switzerland; 3 Psychiatric University Hospital (LMU), 80336 Munich, Germany; 4 Institute for Laboratory Medicine, University of Munich (LMU), 81377 Munich, Germany; 5 Psychiatric University Hospital (KPPP), 8032 Zurich, Switzerland; 6 Psychiatric University Hospital, 69115 Heidelberg, Germany

**Keywords:** Twins, Within-pair concordance, Quantification, Psychopathology patterns, Inflammatory response system, Vulnerability

## Abstract

The concept of twin concordance involves quantifying the resemblance between co-twins in an “objective” and reproducible way. Yet, quantifying resemblance in the case of complex psychiatric traits like schizophrenic disorders leads to methodological problems, as the yes–no dichotomy of diagnostic schemata does not allow one to assess between-subject differences in psychopathology patterns sufficiently accurately. Therefore, we relied on a multidimensional, quantitative concordance measure that provided a high resolution and differentiation when assessing the resemblance of psychopathology patterns. This concordance measure was central to our investigations into the potential link between schizophrenic disorders and aberrancies of the inflammatory response system. Specifically, we aimed to determine the extent to which (1) the observed variation of between-subject psychopathology concordance among 100 schizophrenic patients and (2) the observed variation of within-pair psychopathology concordance among 71 twin pairs can be explained by immunoglobulin M (IgM) levels. To accomplish this goal, we had to “gauge” in a first step the concordance measure’s performance by (1) comparing the psychopathology patterns of 269 index cases suffering from functional psychoses with the respective patterns of the 350 “affecteds” among their first-degree relatives; (2) systematically comparing the psychopathology patterns of 100 unrelated patients with a diagnosis of schizophrenic disorders with each other; and (3) detailing the within-pair concordance of elementary traits among 2734 healthy twin pairs. As to the role of active immune processes in the context of schizophrenic disorders, we found that there exists a 20–30% subgroup of patients for whom aberrancies of the inflammatory response system, as quantified through IgM levels, appeared to be linked to the pathogenesis of schizophrenic disorders (*r* = 0.7515/0.8184, *p* < 0.0001). The variation of within-pair psychopathology concordance among twins with schizophrenic disorders was found to be “explainable” in part by chronically elevated IgM levels (24.5% of observed phenotypic variance; *p* = 0.0434), thus suggesting that monozygotic twins concordant for schizophrenic disorders may possess a less “robust” variant of the inflammatory response system which can more easily be triggered by exogenous factors than the more “robust” variants of discordant pairs. Though the underlying biological mechanisms remain to be detected, our data have cleared the way for an early identification of patients with schizophrenic disorders for whom the inflammatory response system may be a target for therapeutic intervention. Moreover, our results will likely lead to new treatment strategies that involve elements of personalized medicine.

## Background

The concept of twin concordance involves quantifying the resemblance between co-twins in an “objective” and reproducible way. This leads to methodological problems in the case of complex traits, such as psychiatric diagnoses, symptoms, or syndromes, since the yes–no dichotomy of diagnostic schemata does not allow one to assess between-subject differences in psychopathology patterns sufficiently accurately. Therefore, we relied on a multidimensional, quantitative concordance measure that provided a high resolution and differentiation when assessing the resemblance of psychopathology patterns. Our study laid its focus on schizophrenic disorders, a major diagnostic group in psychiatry, and used the above concordance measure as a key tool when exploring the potential link between schizophrenic disorders and aberrancies of the inflammatory response system.

The term “schizophrenia” was introduced more than 100 years ago by Eugen Bleuler, then director of our hospital [1]. The term denotes a group of complex, heterogeneous disorders of diverse etiopathology (“schizophrenic disorders” or, by E. Bleuler: “group of schizophrenias”). Schizophrenic disorders have a prevalence of about 1% in the general population worldwide, virtually independent of socio-cultural and ethnic differences. They cause the loss of the ability to work, to have close relationships, and to have a fulfilling life. Available treatments, though effective, are incomplete as treatment options are non-causal, and there is no cure for the majority of patients (50–60%).

Family studies suggest that schizophrenic disorders aggregate in families but do not segregate, that is, do not follow simple Mendelian modes of inheritance. Evidence for the involvement of genetic factors in the pathogenesis of schizophrenic disorders originated from studies of monozygotic (mz) and dizygotic (dz) twins reared together [e.g., 2]: an average 55% of mz pairs were found to be concordant for schizophrenic disorders, compared to only 15% among the dz pairs, so that the question arises as to why mz co-twins reared in the same environment have a 3.7-fold higher risk to both suffer from schizophrenic disorders than do dz co-twins. The answer to this question is genetics (or paternal epigenetic factors in mz twins only). Yet, these twin studies made it also clear that genetics can explain only part of the etiopathology as 45% of mz twins remain discordant for schizophrenic disorders over a lifetime, even though they share a common genome.

Active immune processes may be relevant for the development of schizophrenic disorders (and major psychiatric disorders in general), as suggested by evidence from recent studies. For example, (1) a population-based study of 7704 patients with a diagnosis of schizophrenic disorders and 192,590 control subjects without psychiatric history has revealed that a parental history of schizophrenic disorders is associated with a fivefold risk for autoimmune diseases, whereas a parental history of autoimmune diseases increased this risk only slightly, by a factor of 1.45 [3]; (2) adiposity and inflammation appear to contribute to the development of depression [4]; (3) several antidepressants and antipsychotics show anti-inflammatory effects [5–11]; (4) several studies demonstrated microglia activation and progressive brain changes in recent-onset schizophrenia [12, 13]; (5) there is postmortem evidence of cerebral inflammation in schizophrenia [14]; (6) significant alterations of T-cell function, along with activation of the inflammatory response system, appeared to be linked to treatment-resistant schizophrenia [3, 15]; and (7) similar processes have been reported for mood disorders as well [16–21].

The abnormalities of CNS metabolism observed with schizophrenic disorders or depression might, therefore, arise because genetically modulated inflammatory reactions damage the microvascular system of the brain, with the nature of the infectious agent being less important than the patients’ genetically influenced inflammatory response [22]. Interestingly, the between-subject variation of the “natural” antibody immunoglobulin M^1^ (IgM) has been found to possess a strong genetic component [23], while chronically elevated IgM levels typically develop years before the first clinical symptoms occur [24].

Moreover, chronically elevated IgM levels appeared to be related to a heritable malfunction in the inflammatory response system [e.g., 23], and may even be linked to autoimmune diseases in general. The pathogenesis of these latter diseases, however, is insufficiently understood, also because the question of autoantibody appearance prior to inflammation—indicating an antibody-driven inflammatory response—could not yet be answered on the basis of empirical data.

To address the question of a potential link between schizophrenic disorders and aberrancies of the inflammatory response, we “gauged” in a first normative step the performance of the multidimensional concordance measure by (1) comparing the psychopathology patterns of 269 index cases suffering from functional psychoses with those of the 350 “affecteds” among their first-degree relatives; (2) comparing the psychopathology patterns of 100 unrelated patients with a diagnosis of schizophrenic disorders with each other; (3) detailing the within-pair concordance of elementary traits among 2734 healthy twin pairs; and (4) comparing the characteristics of the distribution of within-pair IgM concordances with those of elementary traits.

Upon completion of the normative step, we were able to determine the extent to which (1) the observed variation of the between-subject psychopathology concordance among 100 schizophrenic patients and (2) the observed variation of the within-pair psychopathology concordance among 71 twin pairs were explainable by IgM levels. In addition, we searched for evidence supporting the hypothesis that monozygotic twins concordant for schizophrenic disorders possess a less “robust” variant of the inflammatory response system which can more easily be triggered by exogenous factors than the more “robust” variants of discordant pairs.

## Methods

The concept of “concordance” deals with the degree of similarity between the two co-twins of a twin pair and the variation of that similarity across samples of mz and dz twins (“twin method”: used to estimate the genetically predisposed component of a trait). There are numerous approaches to realizing the concept by quantifying within-pair similarities for traits, such as weight, height, brain-wave patterns, personality, behavior, or psychiatric disorders, among others. The simplest way to address within-pair similarity of psychiatric disorders is based on clinical diagnosis and by just counting the number of twin pairs of a sample who share the same diagnosis (“concordance rate”). The problem inherent in this approach lies in the yes/no-dichotomy of diagnostic systems which often means imposing structure on clinical data rather than finding “natural” structure in the data. In the case of schizophrenic disorders, this yes/no-dichotomy can yield concordance rates in the range of 30–70% for the same sample of mz twins depending on the diagnostic system in use. And worst of all, one single item error on the diagnosis level can invert the entire outcome.

Another problem relates to the fact that the yes/no-dichotomy approach to measuring concordance in twins with schizophrenic disorders has never been externally validated or “gauged”. No information is available as to how a rate of “30%” derived through diagnostic system “A” for a given sample of twin pairs compares to the rate of “70%” derived for that sample through diagnostic system “B”; or how a rate of “45%” from hospital “C” compares to the rate of “55%” from hospital “D”. Nor do we know how all this compares to traits, such as height, weight, finger ridge count, brain waves, or behavior.

Quantitative concordance measures avoid these difficulties. They are less error prone when assessing the within-pair similarity of psychopathology among twin pairs with psychiatric disorders. In particular, they provide the methodological framework for (1) more detailed investigations into twins with schizophrenic disorders and (2) the inclusion of resilience factors which are likely to play a critically important role in the pathogenesis of these disorders.

In what follows, we will rely exclusively on quantitative concordance measures, so that we can analyze the distribution of within-pair concordances, can determine potential deviations from normality, and can use the respective mean values and standard deviations as estimates of the genotype–phenotype “distance” inherent in the trait under investigation. We also think that it is absolutely necessary to externally validate and to calibrate a methodological framework prior to applying it to schizophrenia data. Therefore, normative analyses are part of this manuscript.

The psychopathology assessments of this study were based on the syndrome-oriented instruments *SSCL-16* and *SSCL-16 Supplement* which extend the DSM-V and ICD-10 definitions of psychiatric disorders by replacing the yes–no dichotomy of diagnostic schemata by dimensional quantities, thus enabling the inclusion of details of psychopathology patterns that do not reach diagnostic thresholds and would otherwise be ignored. The instruments^2^ were developed by Angst et al. [25] from the lifetime version of the “Schedule for Affective Disorders and Schizophrenia (SADS-L)” [26]. The *SSCL-16* assesses 16 syndromes in a quantitative way through the respective symptoms. Key to schizophrenic disorders are the *SSCL-16* syndromes “schizophrenic thought disorders”, “delusions”, “hallucinations”, “ego consciousness”, “anergia”, “incongruent affect”, “depressive syndrome”, “manic syndrome”, and “attempted suicide”. The *SSCL-16* is complemented by the *SSCL-16 Supplement* which measures the patients’ “overall level of functioning”, “social relations”, “affective lability”, “personality traits”, “somatization”, and “consumption behavior”. Our study was comprised of four independent samples:

1. 269 patients suffering from functional psychoses (schizophrenic-, schizoaffective-, bipolar disorders, and depression with psychotic symptom) along with the 350 “affecteds” among their 1501 first-degree relatives (143 males, 207 females; 124 fathers and mothers with a mean age of 64.6 years; and 226 siblings with a mean age of 34.8 years). This sample was recruited from consecutive admissions to our hospital. The subjects’ psychopathology (lifetime) was assessed through the *SSCL-16* and *SSCL-16 Supplement* instruments by specifically trained interviewers. Details are given in Table 1.
2. 100 patients randomly selected from our databank with an ICD10 diagnosis of schizophrenic disorders (50 males, 50 females). The patients’ psychopathology (lifetime) was assessed through the *SSCL-16* and *SSCL-16 Supplement* instruments by specifically trained interviewers. The patients contributed a 2 × 9 ml blood sample from which DNA and serum were extracted, so that IgM^3^ levels and cytokine data^4^ (*Il-6, TNF-a, Neopterin, TGF-β1*, and *sCD14*) could be measured.
3. 2734 healthy twin pairs^5^ serving as “learning” basis for our investigations into the within-pair concordance of twins with schizophrenic disorders: 1434 monozygotic pairs (592 male pairs, 842 female pairs) with a mean age of 38.3 ± 6.9 years and 1300 same-sex dizygotic pairs (514 male pairs, 786 female pairs) with a mean age of 39.2 ± 6.9 years. The data allowed us to “gauge” the characteristic variation of within-pair concordance measures for elementary traits, such as height, weight, shoe size, finger ridge count, birthweight, among others.
4. Pilot sample of 47 monozygotic and 24 dizygotic same-sex twin pairs. The twins’ psychopathology (lifetime) was assessed through the *SSCL-16* and *SSCL-16 Supplement* instruments by specifically trained interviewers. All subjects contributed a 2 × 9 ml blood sample from which DNA and serum were extracted, so that IgM levels and cytokine data could be measured. Of this sample, 28 pairs had at least one co-twin who received an ICD10 diagnosis of schizophrenic disorders. The other pairs did not meet the criteria of schizophrenic disorders and received either other diagnoses or no diagnosis. Zygosity was determined by a set of five highly polymorphic dinucleotides and confirmed by 448 SNPs.

**Table 1:** Sample composition of the zurich psychosis study regarding index cases (*n* = 269)

	Total	Males	Females	Age (years)	Range (years)
Schizophrenic disorders	140	66	74	35.6 ± 11.7	17–73
Schizoaffective disorders	43	14	29	47.0 ± 12.3	19–69
Bipolar disorders	34	17	17	47.3 ± 15.9	17–72
Depression with psychotic symptoms	52	26	26	55.1 ± 11.3	20–76

### Quantifying within-pair concordance among twins

The most popular concordance measure for single quantitative traits is the Pearson product-moment correlation coefficient which measures the linear correlation between co-twins for a given variable of interest, such as body height, body weight, etc. For a set of *n* monozygotic or dizygotic twin pairs, this correlation coefficient r(*x,y*) is calculated through the following formula (1):

1 $$\begin{aligned} r(x,y)=\frac{1}{{n - 1}}\sum\limits_{i=1}^n {\left( {\frac{{{x_i} - \bar x}}{{{s_x}}}} \right)} \left( {\frac{{{y_i} - \bar y}}{{{s_y}}}} \right)\quad & \bar x=\frac{1}{n}\sum\limits_{i=1}^n {{x_i}} \quad {s_x}=\sqrt {\frac{1}{{n - 1}}\sum\limits_{i=1}^n {{{({x_i} - \bar x)}^2}} } \\ & \bar y=\frac{1}{n}\sum\limits_{i=1}^n {{y_i}} \quad {s_y}=\sqrt {\frac{1}{{n - 1}}\sum\limits_{i=1}^n {{{({y_i} - \bar y)}^2}} } , \\ \end{aligned}$$

where *x* denotes the variable value of the first co-twin and *y* denotes the variable value of the second co-twin. The running index “*i*” indicates that the formula evaluates the variable values *x,y* for the set of *n* twin pairs as an entity. For sufficiently large samples of twin pairs, the between-pair variation along with confidence intervals can be estimated by means of random sampling methods. Here, the correlation coefficient is computed for randomly selected subsamples comprising about 10% of the total sample. This is repeated a large number of times (“bootstrapping”).

Alternatively, the Intraclass Correlation (ICC) can be used to quantify the degree to which co-twins resemble each other in terms of a quantitative trait. The ICC operates on data structured as groups, rather than data structured as paired observations. In consequence, the ICC approach is the perfect method for the assessment of consistency of quantitative measurements made by several different observers measuring the same quantity. The ICC is calculated through the following formula (2):

2 $$\begin{aligned} r({x_1},{x_2})=\frac{1}{{N{s_2}}}\sum\limits_{i=1}^N {({x_{i,1}} - \bar x)} ({x_{i,2}} - \bar x)\quad & \bar x=\frac{1}{{2N}}\sum\limits_{i=1}^N {({x_{i,1}}+{x_{i,2}})} \\ & {s^2}=\frac{1}{{2N}}\left\{ {\sum\limits_{i=1}^N {{{({x_{i,1}} - \bar x)}^2}} +\sum\limits_{i=1}^N {{{({x_{i,2}} - \bar x)}^2}} } \right\}, \\ \end{aligned}$$

where *x*_1_ denotes the variable value of the first co-twin and *x*_2_ denotes the variable value of the second co-twin. The running index “*i*” indicates that the formula evaluates the variable values for a set of *n* twin pairs as an entity. The differences between Pearson and ICC coefficients are marginal for elementary traits, such as height, weight, shoe size, birth weight, or the BMI (in the range of 1–2%). A disadvantage is that the ICC is only rudimentarily supported by the statistics packages SAS and SPSS.

### Multidimensional approach

If several variables have to be evaluated simultaneously, as is the case for schizophrenic disorders quantified through a set of syndromes, the standard choice is the “Euclidean Distance” which measures “discordance”^6^ between co-twins, or between pairs of subjects, through the square root of the sum of squares of variable differences (3):

3 $$d(\overset{\lower0.5em\hbox{$\smash{\scriptscriptstyle\rightharpoonup}$}} {x} ,\overset{\lower0.5em\hbox{$\smash{\scriptscriptstyle\rightharpoonup}$}} {y} )=\sqrt {\sum\limits_{k=1}^n {{{({x_k} - {y_k})}^2}} } \quad \overset{\lower0.5em\hbox{$\smash{\scriptscriptstyle\rightharpoonup}$}} {x} =({x_1},{x_2}, \ldots {x_n})\quad \overset{\lower0.5em\hbox{$\smash{\scriptscriptstyle\rightharpoonup}$}} {y} =({y_1},{y_2}, \ldots {y_n}),$$

where $$\overset{\lower0.5em\hbox{$\smash{\scriptscriptstyle\rightharpoonup}$}} {x}$$ denotes the multidimensional set of variables of the first co-twin and $$\overset{\lower0.5em\hbox{$\smash{\scriptscriptstyle\rightharpoonup}$}} {y}$$ that of the second co-twin. If the variables differ significantly with respect to range and magnitude, the Euclidean Distance has to be “normalized” or “standardized” to avoid situations where one variable “overpowers” all others. Normalization is achieved by dividing each variable’s difference either by the absolute range or the standard deviation of the respective *k*th variable, calculated across all co-twins (4):

4 $$\begin{aligned} {d^n}(\overset{\lower0.5em\hbox{$\smash{\scriptscriptstyle\rightharpoonup}$}} {x} ,\overset{\lower0.5em\hbox{$\smash{\scriptscriptstyle\rightharpoonup}$}} {y} )=\sqrt {\sum\limits_{k=1}^n {\frac{{{{({x_k} - {y_k})}^2}}}{{{s^k}}}} } \quad & {s^k}=\sqrt {\frac{1}{{2n - 1}}\sum\limits_{i=1}^n {\left\{ {{{(x_i^k - {{\bar x}^k})}^2}+{{(y_i^k - {{\bar x}^k})}^2}} \right\}} } \quad k{\text{th}}\;{\text{vector}}\;{\text{component}} \\ & {{\bar x}^k}=\frac{1}{{2n}}\sum\limits_{i=1}^n {(x_i^k+\bar y_i^k)} \quad k{\text{th}}\;{\text{vector}}\;{\text{component,}} \\ \end{aligned}$$

5 $$s(\overset{\lower0.5em\hbox{$\smash{\scriptscriptstyle\rightharpoonup}$}} {x} ,\overset{\lower0.5em\hbox{$\smash{\scriptscriptstyle\rightharpoonup}$}} {y} )=\frac{1}{{d(\overset{\lower0.5em\hbox{$\smash{\scriptscriptstyle\rightharpoonup}$}} {x} ,\overset{\lower0.5em\hbox{$\smash{\scriptscriptstyle\rightharpoonup}$}} {y} )+1}},$$

where “$${d^n}(\overset{\lower0.5em\hbox{$\smash{\scriptscriptstyle\rightharpoonup}$}} {x} ,\overset{\lower0.5em\hbox{$\smash{\scriptscriptstyle\rightharpoonup}$}} {y} )$$” denotes the normalized Euclidean Distance with $$\overset{\lower0.5em\hbox{$\smash{\scriptscriptstyle\rightharpoonup}$}} {x}$$ being the multidimensional set of variables of the first co-twin and $$\overset{\lower0.5em\hbox{$\smash{\scriptscriptstyle\rightharpoonup}$}} {y}$$ that of the second co-twin. The multivariate concordance measure $$s(\overset{\lower0.5em\hbox{$\smash{\scriptscriptstyle\rightharpoonup}$}} {x} ,\overset{\lower0.5em\hbox{$\smash{\scriptscriptstyle\rightharpoonup}$}} {y} )~$$ is derived directly from the Euclidean Distance through formula (5).

### Statistical analyses

Statistical analyses were carried out by means of the Statistical Analysis Software SAS/STAT 9.3 [PROCs FREQ, MEANS, CORR, REG, and GLM (unbalanced data)], while a proprietary program of our institute was used for random sampling and the generation of postscript plots.

The study was approved by the local ethics committees and written informed consent was obtained from all participants. There are no conflicts of interest.

## Results

### Index cases with schizophrenic disorders versus affected first-degree relatives

**Fig. 1:**
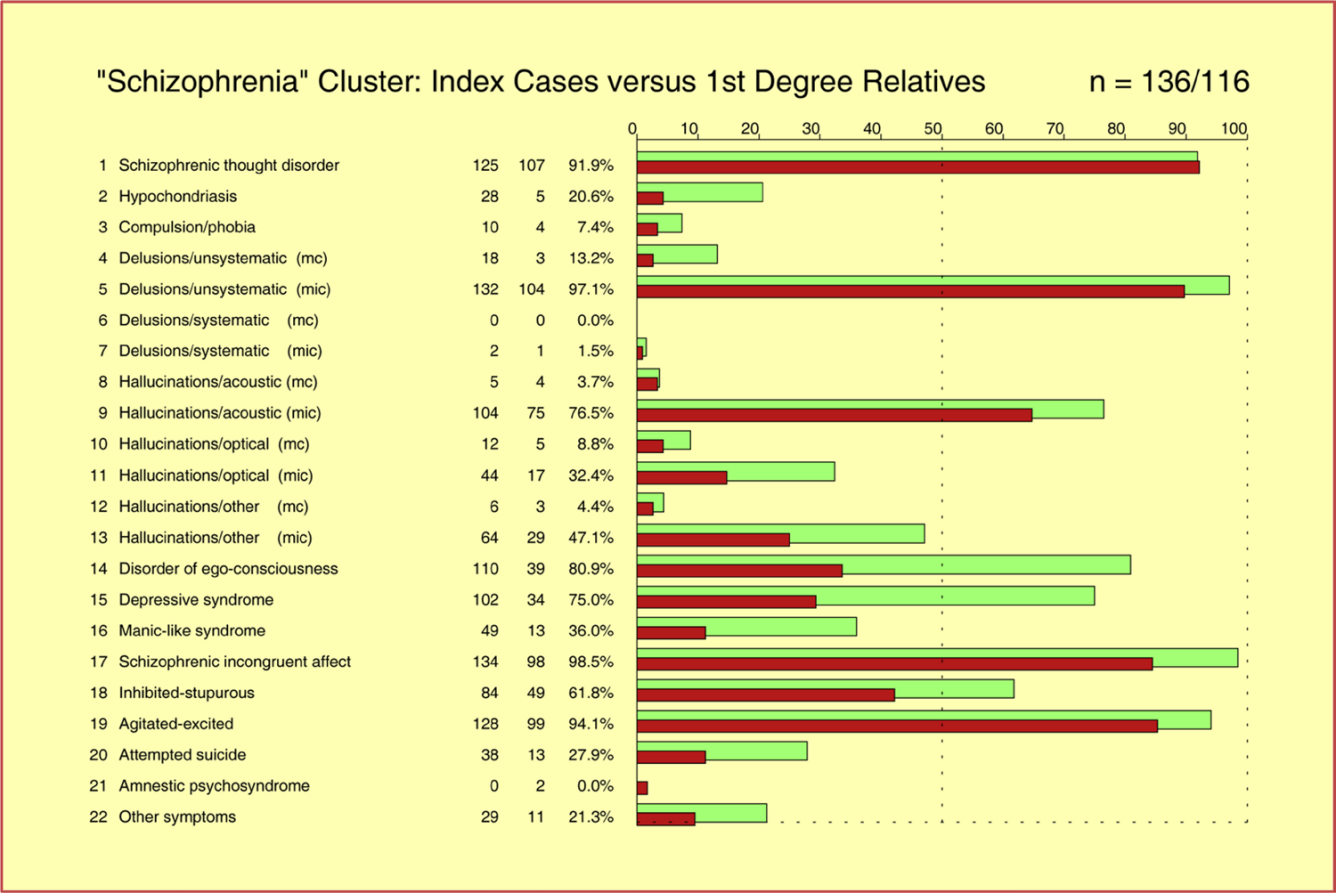
Highly significant similarity (*p* < 0.001) between the SSCL-16 syndrome profiles derived by averaging across index cases of the “Schizophrenia Cluster” (*n* = 136; *green bars*) and the SSCL-16 syndrome profiles derived by averaging across their “affected” first-degree relatives (*n* = 116; *red bars*). On the diagnosis level, by contrast, no homotypal similarity between index cases and first-degree relatives was found in the sense that the index cases’ clinical diagnoses predominantly occur among members of their families (total sample size: 269 index cases along with 350 “affecteds” among their first-degree relatives)

Quite a number of diagnosis-oriented family studies of schizophrenic disorders have demonstrated that there is no homotypal similarity between index cases suffering from schizophrenic disorders and their affected first-degree relatives in the sense that the index case’s clinical diagnosis predominantly occurs among family members, irrespective of the diagnostic schema in use. On the syndrome level, by contrast, our quantitative analysis revealed highly significant similarities (*p* < 0.001) between the syndrome profiles of index cases of the “Schizophrenia Cluster” (*n* = 136) and the syndrome profiles of their affected first-degree relatives (*n* = 116) (Fig. 1).

This finding suggested that studies of schizophrenic disorders which exclusively rely on clinical diagnoses are likely to suffer the loss of critically important information, and may obscure the “true” nature of this group of heterogeneous disorders of diverse etiopathology (E. Bleuler: “group of schizophrenias”).

### Calibration sample of 100 patients with an ICD10 diagnosis of schizophrenic disorders

On the diagnostic level, all possible comparisons between patients with the same psychiatric diagnosis necessarily yield concordances of 100%. By contrast, quantitative psychopathology scores as derived from the *SSCL-16* and *SSCL-16 Supplement* allow one to study the between-patient concordance at high resolution and differentiation, so that the specifics of individual psychopathology patterns are revealed. We formed all *n* × (*n* − 1)/2 = 4950 possible pairings of the patients and calculated the between-patient concordances for several quantitative syndrome profiles that describe schizophrenic disorders at different levels of detail. Special focus was laid on the differences in psychopathology regarding clinical picture, severity of illness, age of onset, and response to treatment. All these aspects can differ greatly between patients despite the fact that all have received the same psychiatric diagnosis.

**Fig. 2:**
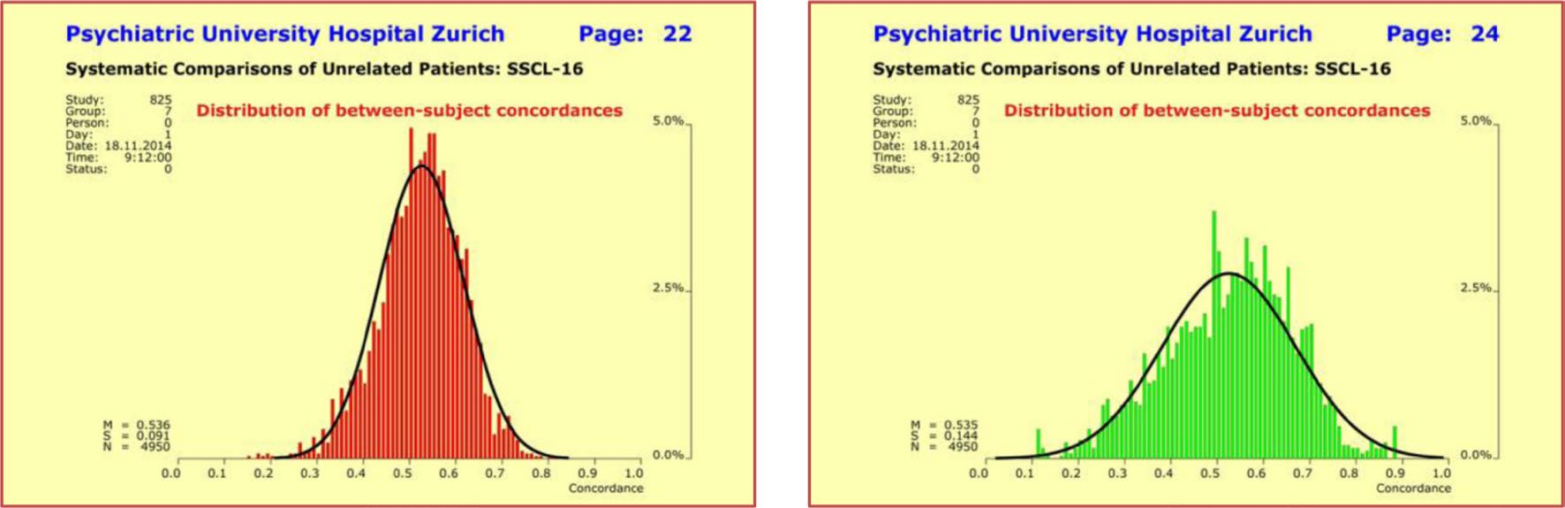
Variation of between-subject concordances of *n* = 100 unrelated subjects with a diagnosis of schizophrenic disorders. Concordances were calculated for all *n* × (*n* − 1)/2 = 4950 possible pairings and for the two combined quantitative SSCL-16 syndromes “schizophrenic thought disorders” and “depressive syndrome” (*upper left*) and for the four combined quantitative SSCL-16 syndromes “schizophrenic thought disorders”, “delusions”, “hallucinations”, and “ego consciousness” (*upper right*). The mean concordance rates were virtually identical, while the range of variation increased with the number of syndromes

Concordance analyses for all syndrome profiles under investigation yielded approximately normal distributions with surprisingly robust and virtually identical concordance rates. The mean values lay around 0.536 ± 0.091 (concordance 53.6%), as long as the key syndromes of schizophrenic disorders were part of the profiles. As one would expect, the more details on clinical picture, severity, onset, and response to treatment were included, the broader the observed range of variation in between-patient concordance (Fig. 2). Most interestingly, the observed between-patient concordances were almost identical with the within-pair concordances reported in the literature for mz twins (55%).

For the sample as a whole entity, our analyses yielded no significant correlations between IgM levels and psychopathology syndrome scores. However, it was readily possible (through the selection of suitable cases) to “construct” a 20–30% subgroup for which significant correlations showed up. A simple IgM cut-off value turned out to be insufficient for this selection. We used instead a standard neural network classifier as a more reliable approach to identifying patients with the highest association between IgM levels on the one hand, and all four scores of the core syndromes of schizophrenic disorders on the other (schizophrenic thought disorders, delusions, hallucinations, and ego consciousness).

The algorithm adjusted the group size while maximizing the IgM-syndrome correlations for the group as an entity. The procedure yielded the following correlations (group size 25, nonparametric Spearman Rank): for “schizophrenic thought disorders” *r* = 0.7515, *p* < 0.0001; for “delusions” 0.5251, *p* < 0.007; for “hallucinations” 0.5320, *p* < 0.006; and for “ego consciousness” *r* = 0.8184, *p* < 0.0001. The other syndrome correlations did not reach statistical significance: “anergia” (*r* = −0.1867), “incongruent affect” (*r* = 0.1478), and “depressive syndrome” (*r* = −0.1270).

None of the cytokines under investigation had a significant influence on the observed IgM levels, thus suggesting that acute infections played a minor role in this patient sample, if at all. In consequence, the combined assessment of *SSCL-16* syndrome scores and IgM levels apparently enables the early identification of that 20–30% subgroup of patients for whom the inflammatory response system might be a target for therapeutic intervention.

### Normative sample of 2734 healthy twin pairs

**Fig. 3:**
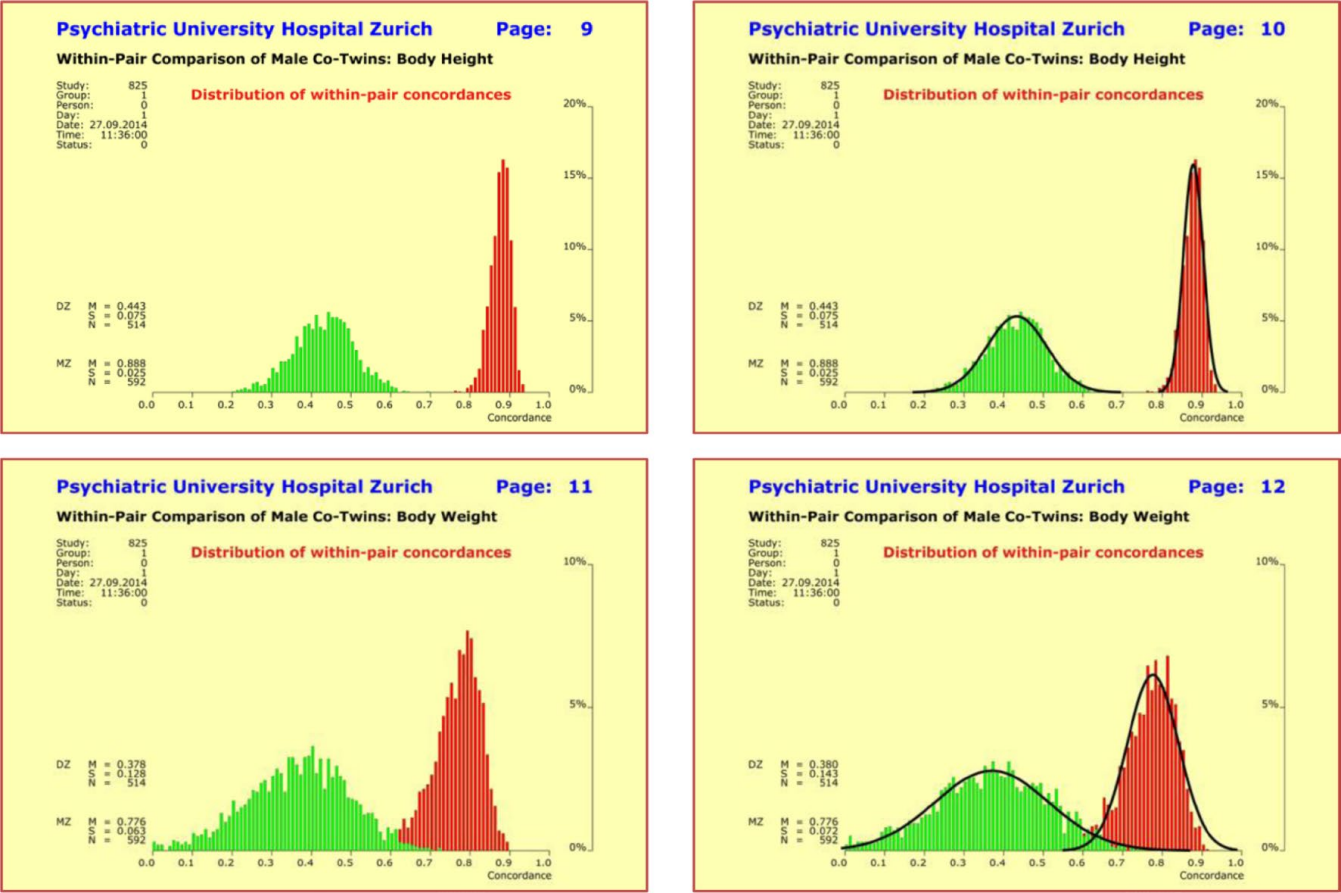
Within-pair concordances of model traits “body height” (*upper half*) and “body weight” (*lower half*) for monozygotic (mz: *red*) and dizygotic (dz: *green*) twins. The distributions were calculated separately for males and females to detect potential gender-related differences, but no such differences were found. The empirical distributions are approximately normally distributed as demonstrated through the black distribution *curves* on the *right side*

As to directly assessable elementary traits, it is interesting to note that monozygotic (mz) and dizygotic (dz) concordances reported in the literature are given as bare mean values. No information is available about the statistical distributions of mz and dz concordances which may be non-normal. To address this issue, we calculated within-pair concordances separately for males and females based on formula (1) in combination with a bootstrapping approach (1000 randomly selected subsamples comprising 5–8% of each subset of twin pairs defined by gender and zygosity). We found, for example, normal distributions for body height and body weight (Fig. 3).

**Fig. 4:**
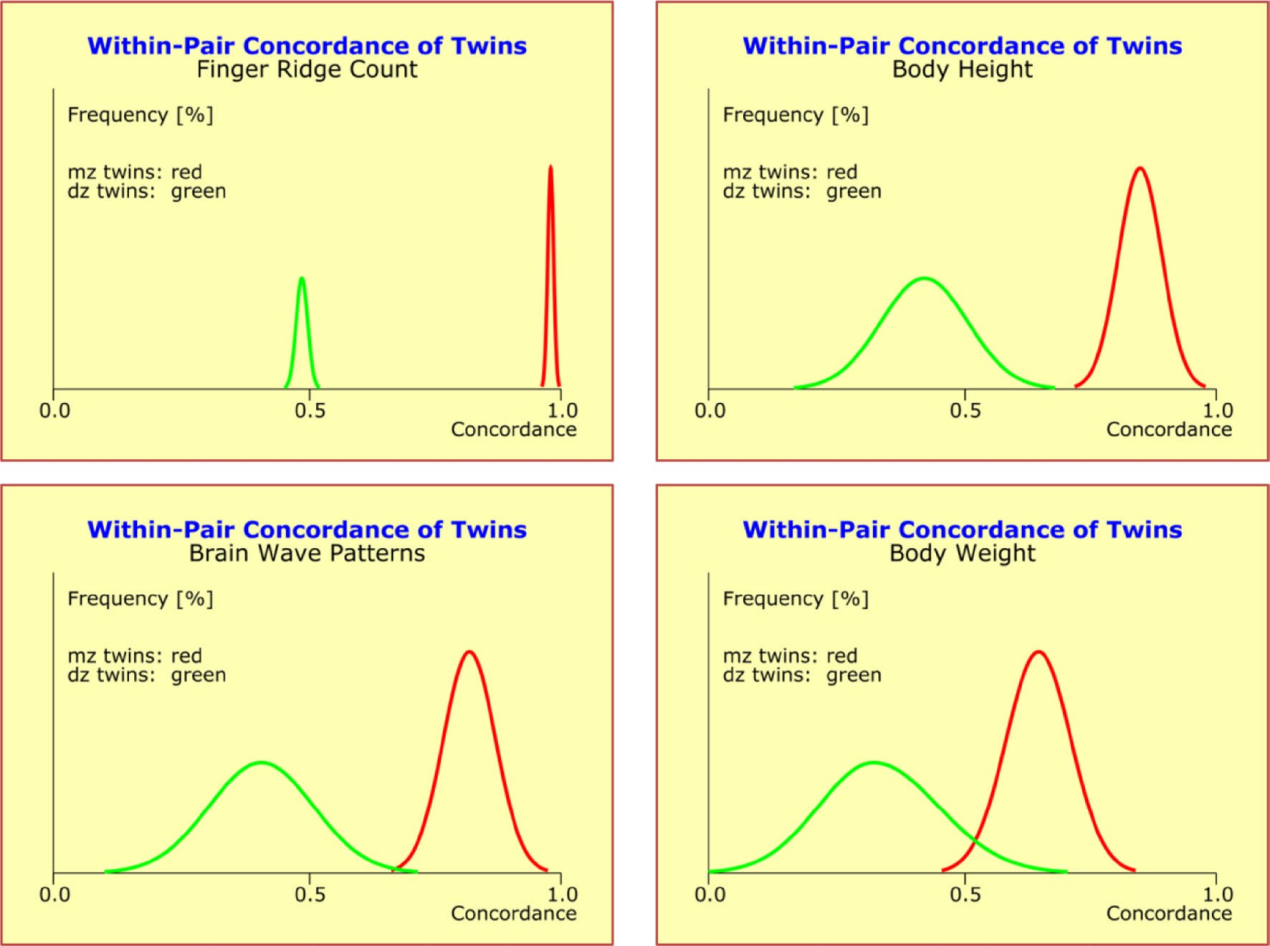
Distributions of the within-pair concordances of the quantitative traits “finger ridge count” (*upper left*), “body height” (*upper right*), “brain wave patterns” (*lower left*), and “body weight” (*lower right*) presented as ideal normal distributions *N*(*µ,σ*) with mean “*µ*” and standard deviation “*σ*”. The distribution curves of monozygotic (mz) twins are in red and those of the dizygotic (dz) twins in green. Differences between traits relate to differences in *µ* and *σ*, with *µ* and *σ* being inversely related to each other: the larger the observed *µ* the smaller the corresponding *σ*. The means of all four traits exhibit a mz:dz ratio of 2:1

In detail, the mean concordance rates were 0.891 (mz; concordance 89.1%) and 0.448 (dz; concordance 44.8%) for variable “body height”, and 0.781 (mz; concordance 78.1%) and 0.388 (dz; concordance 38.8%) for variable “body weight”, respectively. In particular, the mean concordance rates of both variables did not show gender differences and displayed a mz:dz ratio of exactly 2:1 independent of the actual magnitudes. Mean values and standard deviations were inversely related to each other: the larger the mean value the smaller the standard deviation. Using the same method of approach, we subsequently determined the distributions of mz and dz concordances for additional elementary traits. Again, no gender differences were found (Fig. 4).

The numerical results from these analyses are summarized in Table 2. The most unexpected result was that all variables showed a mz:dz ratio of exactly 2:1 indicating polygenic additive modes of inheritance. The variable “birthweight” was the only exception with a mz:dz ratio of 1:1, thus suggesting that environmental factors played a dominant role for this variable and obscured genetic predisposition (Table 2).

**Table 2:** Quantitative traits “finger ridge count”, “body height”, “brain wave patterns”, “shoe size”, and “body weight” display mean concordance rates for monozygotic (mz) and dizygotic (dz) twin pairs which have a mz:dz ratio of 2:1 irrespective of the actual magnitude

	mz twins	dz twins
Within-pair concordance of quantitative traits		
Finger ridge count	0.990	0.500
Body height	0.891	0.448
Brain wave patterns	0.824	0.415
Shoe size	0.875	0.454
Body weight	0.781	0.388
Birth weight	0.695	0.694

The only exception is “birthweight” with an mz:dz ratio of 1:1. Deviations from the expected ratio of 2:1 indicate the existence of strong endogenous or exogenous factors that modify the trait under comparison for both co-twins in likewise ways. Pearson correlation coefficients were chosen, because (1) the differences between Pearson and ICC are marginal for these traits, in the range of 1–2%; and (2) the ICC is only rudimentarily supported by the standard statistics packages SAS and SPSS and is, therefore, not readily available to people interested in verifying these results

Contrary to expectations, we did not find a significantly reduced within-pair concordance in dz twin pairs for the variable “body weight”, despite major environmental challenges over 10–15 years after adolescence, accentuated by the fact that many co-twins lived separated from each other in different environments (with different partners) for quite a number of years. Hence, environmental factors appeared to have a small impact compared to the dominating genetic component.

**Fig. 5:**
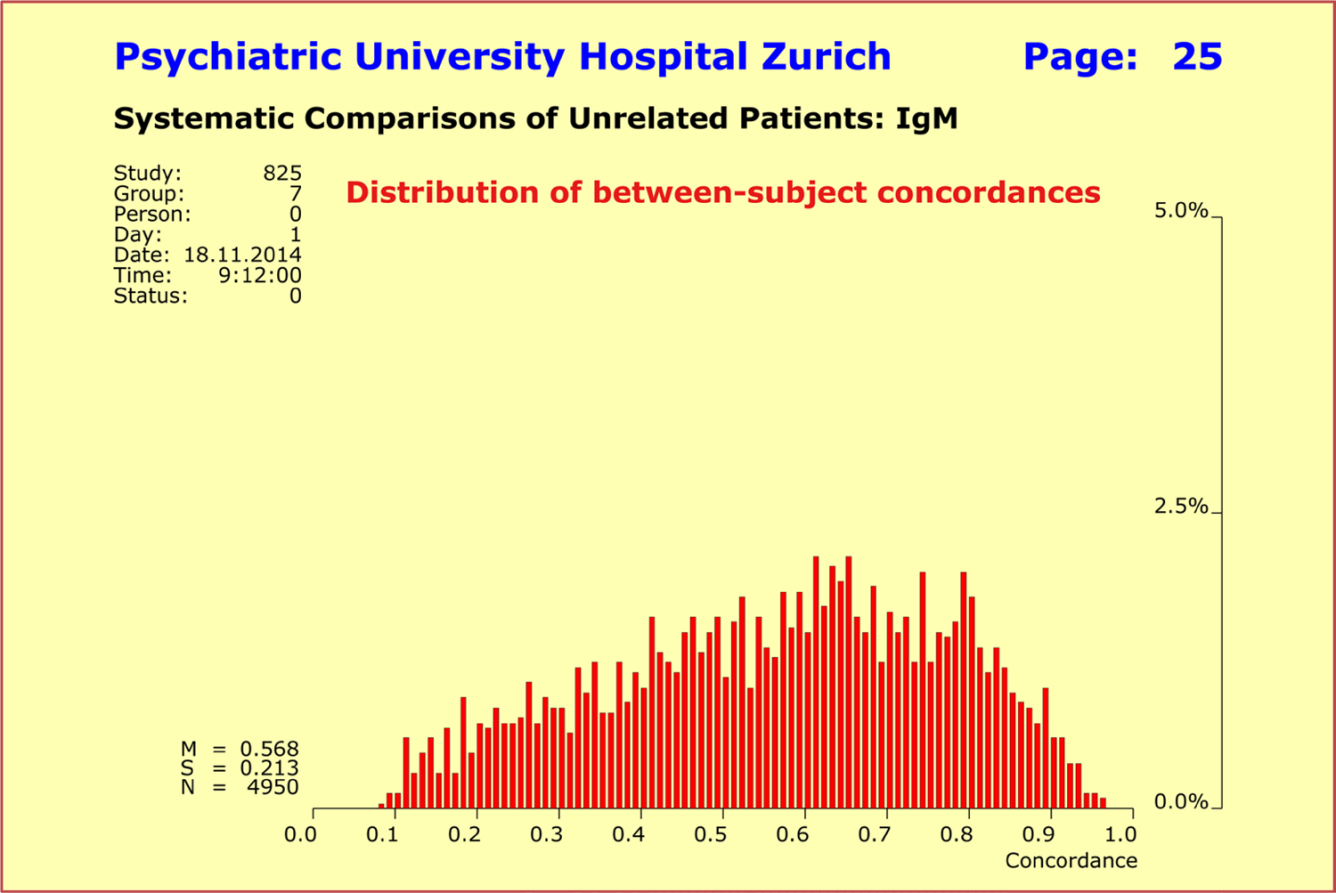
Distribution of between-subject IgM concordances was found to be right-skewed and showed a broad range of variation among the *n* = 100 patients with schizophrenic disorders (total *n* × (*n* − 1)/2 = 4950 possible pairings). The characteristics differed completely from those of the model traits “body height” and “body weight”, thus suggesting the existence of principal differences in the underlying biological mechanisms. The observed skewness was due to the fact that the population encompassed two subgroups: more than 70% of the patients had very low IgM levels, whereas the other 30% of patients exhibited somewhat elevated levels. Therefore, concordances calculated between patients of the same subgroup were higher than those calculated between patients of different subgroups

Once the normative analyses were completed, we calculated the between-patient IgM concordances for all possible pairings of the 100 patients with a diagnosis of schizophrenic disorders. Using the same methodological framework as in the normative analyses (random sampling: Pearson product-moment coefficient with “bootstrapping”), we found a right-skewed distribution whose characteristics differed substantially from those of the model traits “body height” and “body weight”. This finding suggested the existence of principal differences in the underlying biological mechanisms. The observed skewness was due to the fact that the patient population encompassed two subgroups: (1) more than 70% of the patients had very low IgM levels (first subgroup), whereas (2) the other 30% of patients exhibited somewhat elevated levels (second subgroup) with cut-off set to IgM ≥13.5 (cf. [23]). Clearly, concordances calculated between patients of the same subgroup were much higher than those calculated between patients of different subgroups (Fig. 5).

### Pilot sample of twins concordant and discordant for schizophrenic disorders

For schizophrenic disorders, the most frequently reported and generally accepted concordance rates for mz and dz twin pairs show substantial deviations from the mz:dz ratio of 2:1. Specifically, mz co-twins reared in the same environment are reported to have a concordance rate of 0.55 (55%), whereas dz co-twins reared in the same environment are reported to show a concordance rate of only 0.15 (15%). To address the question of the extent to which the variation of within-pair psychopathology concordance is explainable through IgM levels, we applied the multidimensional quantitative concordance measure to our pilot sample of twins concordant and discordant for schizophrenic disorders. In particular, we determined the within-pair concordances of mz and dz twins for the various psychopathology syndrome profiles under investigation as well as for the IgM levels.

**Fig. 6:**
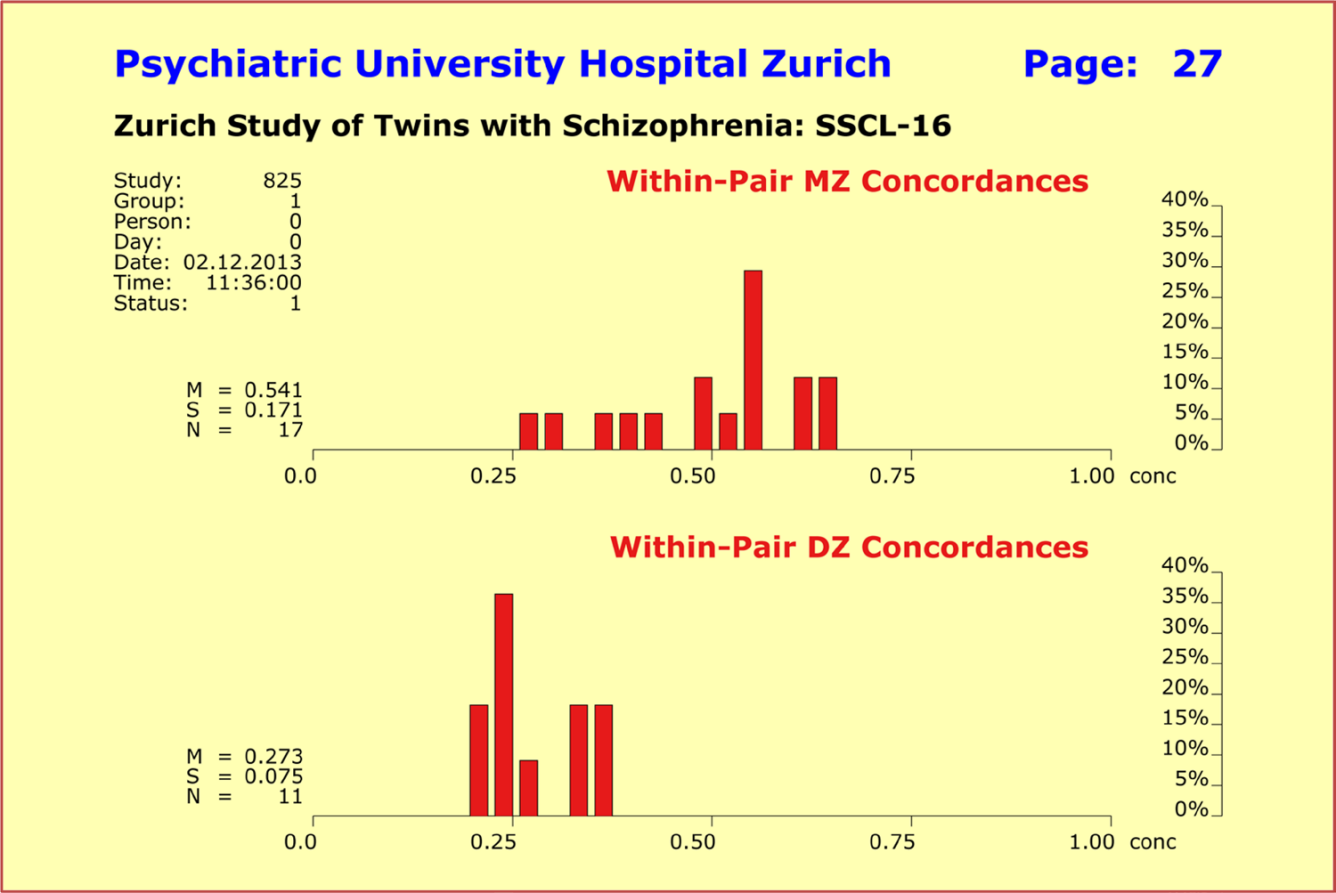
Within-pair concordances in terms of the psychopathology profile made up by the SSCL-16 syndromes “schizophrenic thought disorders”, “delusions”, “hallucinations”, “ego consciousness”, “anergia”, “incongruent affect”, and “depressive syndrome”. We found a mz:dz ratio of 2:1 with mean values of 0.5408 ± 0.1708 for the monozygotic (mz) pairs and 0.2728 ± 0.0752 for the dizygotic (dz) pairs. This mz:dz ratio turned out to be very robust in regard to adding additional syndromes to or removing single syndromes from the profile. The sample sizes are by far too small to reveal the underlying normal distributions

Contrary to expectations, we found a mz:dz ratio of almost exactly 2:1 for the syndrome profiles made up by the *SSCL-16* syndromes “schizophrenic thought disorders”, “delusions”, “hallucinations”, “ego consciousness”, “anergia”, “incongruent affect”, and “depressive syndrome” with mean values of 0.5408 ± 0.1708 (mz pairs; concordance 54.08%) and 0.2728 ± 0.0752 (dz pairs; concordance 27.28%). The mz:dz ratio of 2:1 turned out to be very robust when adding single syndromes to, or removing single syndromes from, the profile (Fig. 6).

The within-pair psychopathology concordances among the mz pairs of our pilot sample varied across the interval (0.281–0.656), that is, exhibited a remarkably broad range from “discordant” to “concordant” without, however, reaching “perfect” discordance or concordance. In fact, there was not a single mz pair in our sample that displayed perfect concordance regarding schizophrenic disorders^7^. The within-pair concordances in terms of IgM levels showed with 0.7480 ± 0.1362 (mz pairs; concordance 74.8%) and 0.5086 ± 0.2750 (dz pairs; concordance 50.86%) significant deviations from the expected 2:1 ratio, thus suggesting that IgM levels, regarded as dynamic system outputs, were at least for some of the twin pairs not in “baseline position”. This in turn suggested the existence of bodily responses to or interactions with endogenous or exogenous factors (as was the case with variable “birthweight”).

Finally, we applied a General Linear Model (GLM) to the mz pairs’ psychopathology and IgM data to estimate the extent to which the variation of within-pair psychopathology concordance among mz twins with schizophrenic disorders was “explainable” through chronically elevated IgM levels. Syndrome profiles were made up again by the SSCL-16 syndromes “schizophrenic thought disorders”, “delusions”, “hallucinations”, “ego consciousness”, “anergia”, “incongruent affect”, and “depressive syndrome”. The fitted GLM (simple regression) used “IgM level” as independent and “within-pair concordance” as dependent variable. The variable “sex” was removed from the model due to nonsignificant influence (*p* = 0.163). The fitted model included all 17 mz pairs with at least one co-twin suffering from schizophrenic disorders, reached statistical significance (*p* = 0.0434; *F* = 4.87/1 DF), and explained 24.5% of the observed phenotypic variance. The IgM intercept was 0.505 (*p* < 0.0001; *t* = 12.45/1 DF). No such model was found for the dz pairs.

## Discussion

Evidence from an increasing number of studies has suggested that active immune processes are likely involved in the pathogenesis of schizophrenic disorders, or even psychiatric disorders in general [4–14, 17, 18, 23, 27–30]. This raised the intriguing question of whether or not the differences between mz twins concordant and mz twins discordant for schizophrenic disorders might be linked to the “robustness” of the inflammatory response system. This in the sense that mz co-twins concordant for schizophrenic disorders possess a less “robust” variant of the inflammatory response system that can more easily be triggered by endogenous and exogenous factors than the more “robust” variants of discordant pairs.

To accomplish this goal, we replaced the yes–no dichotomy of the diagnostic schemata by dimensional quantities, that is, we replaced the uninformative “100%” concordances on the diagnosis level by multidimensional quantities. This quantitative approach allowed us to analyze inter-individual psychopathology differences among patients with the same clinical diagnoses. In fact, details of psychopathology patterns that did not reach diagnostic thresholds would have been ignored otherwise.

Our results suggested that perfect concordance (100% identity) is a theoretical concept rarely ever observed among patients with the same psychiatric diagnosis or among pairs of mz twins. Rather, quantitative traits show approximately normal distributions *N*(*µ,σ*) with mean “*µ*” and standard deviation “*σ*”, such that 65% of the twin pairs exhibit concordances in the range of *µ* ± *σ* and 95% in the range of *µ* ± 2*σ*. Differences between traits are solely reflected by differences in *µ* and *σ*, with *µ* and *σ* being inversely related to each other: the larger the observed *µ* the smaller the corresponding *σ*. In addition, typically, the more details of a complex trait are covered by a multidimensional concordance measure the smaller the observed *µ*.

Almost identical concordance rates for between-patient and within-pair mz twin comparisons indicated that we were dealing with an average “random” sample of mz twins suffering from schizophrenic disorders—without selection bias towards more concordant or more discordant pairs. In consequence, the epidemiology of within-pair psychopathology concordance among mz twins seems to be best described by a normal distribution, as shown in Fig. 2. Moreover, we expect that such “normal” psychopathology distributions apply for twins with psychiatric disorders in general, with higher concordance rates when twins are ascertained through more severe forms of psychiatric disorders. Taken together, this allows us to conjecture that the genetics involved in schizophrenic disorders acts in the sense of an unspecific vulnerability which is neither a necessary nor a sufficient condition for developing the illness.

This study provided two independent lines of evidence suggesting that genetically influenced aberrancies of the inflammatory response system contribute to this unspecific vulnerability, at least in a 20–30% subgroup of patients suffering from schizophrenic disorders. Our findings from the calibration sample of 100 patients with an ICD10 diagnosis of schizophrenic disorders might have cleared the way for an early identification of patients for whom the inflammatory response system is a target for therapeutic intervention [31]. Once successfully established, the proposed method of approach will enable new treatment strategies involving elements of personalized medicine. This is of major interest for patients, psychiatrists, and other health care providers as currently available treatment options offer only modest efficacies and, most importantly, do not allow one to make any predictions of whether or not a particular patient will respond to a particular treatment or will experience certain unwanted side effects. Given the study results, an estimated 20–30% of patients with schizophrenic disorders may benefit from such new treatment strategies.

This study complemented two previous twin studies where co-twins concordant for schizophrenic disorders exhibited a significantly reduced—rather than increased—within-pair concordance for brain wave patterns. This unexpected finding had led to the hypothesis that nongenetic pathologic processes alter the co-twins’ genetically identical brains in such a way that pathologically different functional changes can develop in the co-twins [32, 33]. It remains an open question, however, whether these functional changes can be linked to aberrancies of the inflammatory response system.

The pilot sample of 47 mz and 24 dz same-sex twin pairs was by far too small to draw definitive conclusions regarding the extent to which active immune processes are involved in the pathogenesis of schizophrenic disorders. In particular, the question of whether or not the differences between mz twins concordant and mz twins discordant for schizophrenic disorders might be linked to the “robustness” of the inflammatory response system could not be answered in a definite way. Comorbid disease processes might explain our findings as well.

Another limitation relates to the fact that psychopathology assessments on family members of index cases typically yield a less severe clinical picture compared to that of the acutely ill and hospitalized index cases. Most family and twin studies show this phenomenon which is likely an artifact due to the differences in the amount and quality of available information on psychiatric episodes in the past. However, the possibility of a “true” effect of this kind cannot be ruled out on the basis of empirical data. Similarly, the observation that females were more likely to participate in our studies than males (both index cases and relatives) must not necessarily have introduced an unwanted bias into the data but, rather, may be an intrinsic property of schizophrenic disorders.

## Conclusions

Using a study design with four independent samples, our results provided two independent lines of evidence for an involvement of the inflammatory response system in the pathogenesis of schizophrenic disorders: (1) there existed a 20–30% subgroup of patients for whom aberrancies of the inflammatory response system appeared to be linked to the pathogenesis of schizophrenic disorders (*r* = 0.7515/0.8184, *p* < 0.0001) and (2) the variation in mz concordance regarding schizophrenic disorders was in part “explainable” through chronically elevated IgM levels (24.5% of observed phenotypic variance; *p* = 0.0434). Though the underlying biological mechanisms remain to be detected, our data have cleared the way for an early identification of patients with schizophrenic disorders for whom the inflammatory response system may be a target for therapeutic intervention. We expect that this finding will lead to new treatment strategies that have their focus on a personalized medicine.

## Footnotes

1 IgM autoantibodies are proteins produced by the immune system in response to a perceived threat—which also may be the result of an aberrant immune response of the organism against its own cells and tissues (autoimmune inflammatory processes).

2 Available on website “http://www.bli.uzh.ch/Left07b.php”.

3 IgM levels were determined by the Viollier Laboratories, Basel.

4 Data courtesy Müller N. and Schwarz M.

5 Data courtesy David T. Lykken.

6 “Concordance” and “discordance” are inversely related to each other.

7 This is different from the qualitative diagnosis-oriented approach where co-twins who both meet the diagnostic threshold are deemed to be perfectly concordant (100%) irrespective of differences in their psychopathology pattern. Analogously, if one co-twin does not meet the diagnostic criteria and the second co-twin does, the pair is treated as perfectly discordant.
